# Prevalence and Characteristics of *mcr-1-*Producing *Escherichia coli* in Three Kinds of Poultry in Changsha, China

**DOI:** 10.3389/fmicb.2022.840520

**Published:** 2022-04-07

**Authors:** Jufang Hu, Jie Yang, Wenxin Chen, Zhihong Liu, Qin Zhao, Hui Yang, Zhiliang Sun, Xiaojun Chen, Jiyun Li

**Affiliations:** ^1^College of Veterinary Medicine, Hunan Agricultural University, Changsha, Hunan, China; ^2^Hunan Engineering Technology Research Center of Veterinary Drugs, Hunan Agricultural University, Changsha, Hunan, China; ^3^Liuyang Animal Disease Prevention and Control Center, Hunan, China

**Keywords:** poultry, quail, laying duck, broiler, colistin, *mcr-1*, *Escherichia coli*

## Abstract

Colistin is one of the last-line drugs against difficult to treat and multidrug-resistant Gram-negative bacteria. The emergence of mobile colistin resistance gene m*cr-1* increased worldwide attention on colistin resistance. *mcr-1* is the dominant gene that caused resistance to colistin in chicken-derived *Escherichia coli* (*E. coli*) in China; it has a broad resistance spectrum and causes multiple drug resistance problems. There are only few studies on *mcr*-positive *E. coli* (MCRPEC) from laying ducks and quails in China. Here, the molecular and epidemiological characteristics of MCRPEC from three kinds of poultry farms (laying duck, quail, and broiler) were investigated in Changsha, China. A total of 17 *mcr*-positive *E. coli* (MCRPEC) strains were screened in 690 samples from the three kinds of poultry farms. This is the first report on MCRPEC, to our best knowledge, derived from quail. All the MCRPEC strains were resistant to colistin, sulfamethoxazole-trimethoprim, florfenicol, tetracycline, and ciprofloxacin, and mildly resistant to tigecycline, gentamicin, piperacillin/tazobactam, cefotaxime, and ceftiofur. All the strains were sensitive to meropenem and amikacin. By bioinformatics analysis, 17 MCRPEC strains belonging to 11 MLST types were distributed in phylogroups A (58.8%), B1 (23.5%), and phylogroup D (17.6%). *mcr-1* was located in IncI2 plasmid with typical plasmid conjugation transfer part, type IV secretory system, and tellurium-resistant protein, increasing transmission capacity to other bacteria. Monitoring of colistin-resistant bacteria in poultry farms should be strengthened.

## Introduction

The use of colistin as an antibiotic for the treatment of Gram-negative infections has been gradually reduced because of its nephrotoxicity and neurotoxicity (Koch-Weser et al., 1970). With the emergence of multi-drug-resistant bacteria, especially carbapenem-resistant bacteria, lack of new antibiotics against Gram-negative pathogens has forced the reuse of traditional antibiotic (colistin) (Falagas and Kasiakou, 2005). However, it will be decreasing effectiveness of colistin in clinical after the emergence of mobile colistin resistance gene, *mcr-1*(Liu et al., 2016), and its variants (*mcr-2* to *mcr-10*) (Xavier et al., 2016; AbuOun et al., 2017; Borowiak et al., 2017; Carattoli et al., 2017; Yin et al., 2017; Wang X. et al., 2018; Yang et al., 2018; Carroll et al., 2019; Wang C. et al., 2020). Colistin-resistant isolates harboring these plasmid-mediated colistin resistance genes had been increasing on human clinical (Li et al., 2021), livestock and poultry farms (Hassen et al., 2020), sewage treatment systems (Yang et al., 2019), meat products retail (Sadek et al., 2021), waterfowl breeding (Shen et al., 2019), and home water purifiers (Chen et al., 2021). *mcr* genes have been mainly found in *Escherichia coli*, *Klebsiella pneumoniae*, *Salmonella enterica*, *Enterobacter* spp., and *Aeromonas* spp. (Ling et al., 2020). *mcr-1* is the most widely disseminated plasmid-mediated colistin-resistant gene and is mainly carried by the IncI2 and IncX4 types of plasmid (Tang et al., 2021).

The World Organization for Animal Health (OIE) recommends that organizations urgently prohibit colistin use as a growth promoter because of increasing colistin resistance.^1^ At the same time, governments began to issue policies to limit colistin in livestock and poultry. The Chinese government formally banned colistin as an animal growth promoter on April 30, 2017 (Walsh and Wu, 2016). Other countries, including India, Japan, Malaysia, and Thailand, have banned or agreed to ban colistin as a feed additive for animal growth (Olaitan et al., 2021). The withdrawal of colistin from animal feed in China has significantly reduced colistin resistance and the prevalence of *mcr-1* in both animals and humans (Wang Y. et al., 2020). It proved that the intervention policies effectively reduced the use of colistin and reduced colistin resistance in animals.

*mcr-1* and its variants have been found in various countries and regions but mainly focused on livestock breeding, especially pig and broiler chickens. The prevalence of *mcr-1* in other kinds of poultry is rare. Here, we investigated the prevalence and characteristics of *mcr*-positive *Escherichia coli* (MCRPEC) isolate from three types of poultry farms, namely, quail farm, laying duck farm, and broiler farm, in Changsha, China.

## Materials and Methods

### Sample Collection and Isolation of Bacteria

From May 2019 to May 2020, animal cloacal swabs and surrounding environmental samples were collected from three kinds of poultry farms in Changsha as previously described (Wang et al., 2017; Shi et al., 2021; Zhao et al., 2021). Briefly, 50 animal cloacal swabs per farm were collected using sterile swabs and then suspended in 1 ml phosphate-buffered saline (PBS). Flies were caught using fly glue boards (Green Leaf Co., China) and transferred to the 1 mL PBS with sterile tweezers. Four ml of drinking water and sewage were collected in a clean bottle. Around the farming area, surface soil of 5–10 cm was removed with a shovel, and 5-g soil samples were collected with sterile bags. Five samples from within one square meter were mixed. All the samples were stored in an icebox and transported to the laboratory. The samples were seeded in MacConkey (Solarbio) containing 2 mg/L of colistin and incubated at 37°C overnight; Then, a single pink clone was picked up.

### Screening for *mcr* Genes and Species Identification

In all the bacteria, we detected the presence of *mcr-1* to *mcr-10* by polymerase chain reaction (PCR) (Rebelo et al., 2018; Borowiak et al., 2020; Wang C. et al., 2020). Species identification of MCRPEC was confirmed with the 16S rRNA gene (Srivastava et al., 2008). PCR products were sequenced by Tsingke Biological Technology (Changsha, China) using the sanger sequencing method and compared to the GenBank database.

### Antimicrobial Susceptibility Testing and Plasmid Conjugation Assay

As recommended by Clinical & Laboratory Standards Institute (CLSI) M100-S30,^2^ CLSI VET01-A4 (see text footnote 2), and the European Committee on Antimicrobial Susceptibility Testing (EUCAST),^3^ minimum inhibitory concentrations (MICs) of colistin were evaluated by broth microdilution, and 11 antibiotics (meropenem, amikacin, florfenicol, sulfamethoxazole-trimethoprim, piperacillin-tazobactam, tetracycline, ciprofloxacin, gentamicin, tigecycline, cefotaxime, and ceftiofur) were determined with the agar dilution method. *E. coli* ATCC 25922 was used as a quality control strain. The transferability of *mcr-1* was determined by plasmid conjugation assay; streptomycin-resistant *E. coli* C600 serves as the recipient and MCRPEC as the donor strains. Their transconjugants were selected on MacConkey agar with colistin (2 mg/L) and streptomycin (500 mg/L), and confirmed by ERIC-PCR fingerprinting (Amin et al., 2020) for *E. coli* C600 and PCR analysis for the *mcr-1* gene.

### Whole-Genome Sequencing of MCRPEC

Genomic DNA was extracted from the MCRPEC strains using a TIANamp Bacteria DNA kit (Tiangen Biotech Company, China) according to the manufacturer’s instructions, and 150-bp paired-end reads were generated with a HiSeq X Ten platform (Illumina). Bacterial genome assembly was performed with the SPAdes software (Bankevich et al., 2012).

### Bioinformatics Analysis

Multilocus sequence typing (MLST) was performed, antimicrobial-resistant genes were determined by SRST2 (Inouye et al., 2014), and *E. coli* phylogroups were identified with the ClermonTyping scheme (Beghain et al., 2018). The clonal relationship of MCRPEC was evaluated by core genome alignments and with phylogenetic trees, which were constructed with Parsnp (Treangen et al., 2014) and visualized with the online tool iTOL.^4^ Plasmid comparisons of the isolates were performed with BLAST Ring Image Generator (BRIG) (Alikhan et al., 2011), and EasyFig 2.2.5 was used for gene-environment analysis (Sullivan et al., 2011).

## Results

### Detection of MCRPEC

Six hundred ninety samples (250 broiler chicken cloacal swabs, 150 laying duck cloacal swabs, 150 quail cloacal swabs, and 140 environmental samples) were collected from 10 poultry farms in Changsha (Supplementary Table 1). After screening, 17 strains were positive for *mcr-1* (Table 1), but no other *mcr* variants were detected in this study, indicating that *mcr-1* was the dominant *mcr* gene in poultry farms in Changsha. Among the three kinds of poultry breeding, the isolation rate of *mcr-1*-positive MCRPE was highest in broiler (5.6%, 14/250), followed by quail (0.7%, 1/150), and laying duck (0.7%, 1/150) (Table 1). In addition, MCRPEC was detected in the surrounding environment (flies, 1.8%, 1/56) (Table 1). MCRPEC was present in five broiler farms, and detection rate was higher than 2% (2, 4, 4, 8, and 10%, respectively), indicating that urgent attention is needed in broiler breeding.

**TABLE 1:** Isolation rate of *mcr*-positive *Escherichia coli* (MCRPEC) from different sources.

Source	Samples	MCRPEC	Farm name (Number of MCRPEC)
Broiler	250	5.6% (*n* = 14)	Liuyang 1 (4); liuyang 4 (1);liuyang 6 (2); liuyang 7 (5); liuyang 8 (2)
Laying	150	0.7% (*n* = 1)	Liuyang 1 (1)
Duck			
Quail	150	0.7% (*n* = 1)	Changshaxian 1 (1)
Fly	56	1.8% (*n* = 1)	Changshaxian 2 (1)
Soil	44	0	–
Sewage	20	0	–
Drinking water	20	0	–
Total	690	2.5% (*n* = 17)	–

### Antimicrobial Susceptibility Profiles and Antimicrobial-Resistant Genes of MCRPEC

**FIGURE 1:**
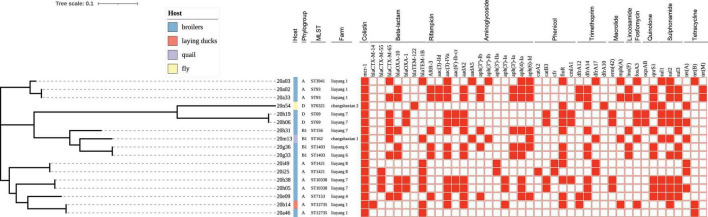
Phylogenetic tree of the 17 *mcr*-positive *Escherichia coli* (MCRPEC) isolates. Phylogroup, multilocus sequence type, and farm origin are shown in text after the corresponding isolate name. Sources of the isolates are differentiated by color. Blue, broiler; Orange, laying duck; Purple, quail; Yellow, fly. Filled squares denote the resistance genes for presence and empty squares for absence.

The MIC range of poultry MCRPEC against colistin was 4 to >128 mg/L. All the MCRPEC isolates were resistant to sulfamethoxazole, florfenicol, tetracycline, and ciprofloxacin, and sensitive to meropenem and amikacin. We found that some of the MCRPEC strains were tetracycline-resistant, but no tetracycline-resistant gene was detected (Figure 1), which may be because of overexpression of the efflux pump gene. In addition, most of the strains were mildly resistant to tigecycline, gentamicin, piperacillin-tazobactam, cefotaxime, and ceftiofur (Table 2 and Supplementary Table 2). Interestingly, different β-lactam-resistant genes (*bla*_CTX_, *bla*_OXA_, and *bla*_TEM_) were found in the 17 MCRPEC strains.

**TABLE 2:** Minimum inhibitory concentration of 11 antibiotics against MCRPEC.

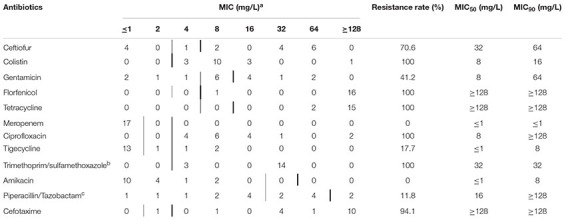

*^a^Thin vertical lines represent the breakpoints between susceptible and insusceptible isolates. Bold vertical lines represent the breakpoints between non-resistant and resistant isolates.*

*^b^Trimethoprim: sulfamethoxazole at a ratio of 1:19. Breakpoints are expressed as trimethoprim concentration.*

*^c^For susceptibility testing purposes, the concentration of tazobactam was fixed at 4 mg/L.*

### Molecular Typing and Phylogenetic Tree

A total of 11 MLST types (ST10338, ST1403, ST1421, ST156, ST162, ST3941, ST6321, ST69, ST7153, ST93, and ST12735) were identified among the 17 MCRPEC strains (Figure 1). MCRPEC isolates predominantly belonged to phylogroup A (58.8%, 10/17), followed by phylogroup B1 (23.5%, 4/17) and phylogroup D (17.6%, 3/17) (Figure 1). Strain 20m13, belonging to ST162, was isolated from a quail farm. To the best of our knowledge, this is the first report on detection of *mcr-1* from quail. In another quail farm, one MCRPEC strain (20n54) from a fly belongs to ST6321 ClermontTypingD *E. coli*, which contains seven resistant genes, namely, β-lactam-resistant gene (*bla*_TEM–122_), tetracycline-resistant gene [*tet*(A)], colistin-resistant gene (*mcr-1*), phenicol-resistant gene (*floR*), fluoroquinolone-resistant gene (*qnrS1*), trimethoprim-resistant gene (*dfrA10*, *dfrA14*), and sulfonamide-resistant gene (*sul1*), which pose a potential risk to quail’s health by spreading to quails.

By constructing a phylogenetic tree, Five groups, with two strains per group isolated from the same farm and closely related, possessed a similar ST type, phylogroup (Figure 1). In addition, two isolates (20a46 and 20b14) that we found in the proximity of the duck and broiler farms had a close genetic relationship (Figure 1).

### Transferability of MCRPEC and Genetic Environment of the *mcr-1* Gene

**FIGURE 2:**
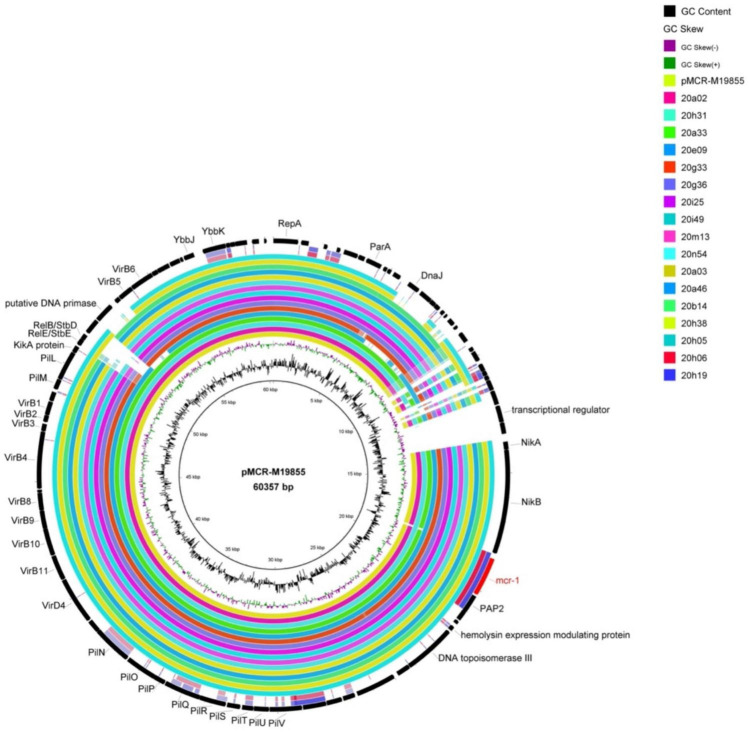
Comparison of whole-genome sequences of MCRPEC against the sequence of *mcr-1*-carrying plasmid pMCR-M19855 (GenBank accession no. KY471315). The first ring from the inside and the coordinates correspond to pMCR-M19855. Each ring represents one of the genomes, and the outside ring represents the regions from the 17 MCRPEC strains in this study.

*mcr-1*-bearing plasmid of most strains (82.4%, 14/17) could transfer to streptomycin-resistant *E. coli* C600 by conjugation assay. By bioinformatics analysis, type IV secretion systems (*virB1*, *virB2*, *virB4*, *virB8*, *virB10*, and *virB11*) and plasmid conjugation elements (*pilM*, *pilV*, and *pilL*) were found in almost all the isolates, which are related to the transferability of bacteria and contribute *mcr-1* to other bacteria (Figure 2). Besides, 70.6% (12/17) *mcr-1*-bearing plasmids belonged to Incl2 except for the additional five contigs whose plasmid type was difficult to distinguish because of the short sequence. Therefore, we speculated that the Incl2 plasmid was prevalent in local poultry farms. Studies show that IS*Apl1* has been deemed to be the critical element mediating the translocation of *mcr-1* into various plasmid backbones (Sun et al., 2017*).* However, IS*Apl1* was not found in the 17 isolates, showing a more stable *mcr-1* genetic environment (Supplementary Figure 1). In addition, this study also found that some of the strains carry tellurium-resistant proteins (TerA, TerB, and TerD), which are involved in tellurite resistance, colicine resistance, phage inhibition, and pathogenicity (Turkovicova et al., 2016).

## Discussion

In this study, colistin was not used in the poultry farms, but the detection rate of the MCRPEC isolates in the poultry farms, especially in the broiler farms, was relatively high. By inquiry, antibiotics are used extensively to promote growth in the broiler; on the contrary, laying ducks are prohibited from using antibiotics during the egg-producing period, because the addition of antibiotics will give rise to veterinary drug residues and reduce the output of egg production. In addition, co-carrier essential resistance genes (*sul*, *floR*, *tet*(M), *bla*, *aph*, etc.) may be related to using other antibiotics (florfenicol, enrofloxacin, doxycycline), along with *mcr-1* which had the potential risk of multi-drug resistant bacteria. Another study showed that *mcr-1*could co-transfer with the *bla*_CTX–M_, *fosA3*, *oqxAB*, and *floR* genes (Sun et al., 2017). Therefore, local regulatory authorities should strengthen the supervision of antibiotic usage and monitor bacterial resistance, especially to MCRPEC strains.

Only a strain from laying ducks (farm liuyang 1) had a close relationship with one MCRPEC strain and a high *mcr-1* detection rate (8%, 4/50) in the broiler farm. Two farms are located in different parts of the same land and managed by the same breeder, which is a potential for transmission from broilers to laying ducks. MCRPEC strains with Incl2 plasmid carrying the type IV secretion system and plasmid conjugation elements promote their *mcr-1* transfer to streptomycin-resistant *E. coli* C600. In this way, it makes the Incl2 plasmid become the dominant carrier of *mcr-1*.

To the best of our knowledge, this study is the first to report on the detection of *mcr-1* on quails. Before this, *Salmonella enterica* carrying *tet*A, *tet*B, and *tet*G (Bacci et al., 2012), and multidrug-resistant methicillin-resistant *Staphylococcus aureus* (LA-MRSA) CC398 carrying the *bla*Z, *mec*A, *erm*B, and *erm*C genes (Silva et al., 2021) were found on quails. There was no significant relationship between the *E. coli* isolated from quail farms and that from other poultry farms (Figure 1). At the same time, MCRPEC from flies in the quail farms was determined, increasing the transmission probability of *mcr-1* in quails (Wang et al., 2017).

We demonstrated the prevalence of MCRPEC isolates in poultry farms in Changsha, but only *mcr-1* gene was found. Poultry MCRPEC shows multi-drug resistance when co-harboring with other essential resistant genes. Likewise, comprehensive genomic analyses revealed that the *mcr-1* genes located in the IncI2 plasmid might transfer to a different host, with the transfer element posing a potential risk to humans. Monitoring of colistin-resistant bacteria in poultry farms should be strengthened.

## Data Availability Statement

The datasets presented in this study can be found in online repositories. The names of the repository/repositories and accession number(s) can be found in the article/Supplementary Material.

## Author Contributions

JL and XC designed the research. JH, QZ, ZL, and HY collected the data. JH, JY, and WC analyzed and interpreted the data. JH drafted the manuscript. JL, JH, ZS, and XC revised the manuscript. All the authors read and approved the final version of the manuscript.

## Conflict of Interest

The authors declare that the research was conducted in the absence of any commercial or financial relationships that could be construed as a potential conflict of interest.

## Publisher’s Note

All claims expressed in this article are solely those of the authors and do not necessarily represent those of their affiliated organizations, or those of the publisher, the editors and the reviewers. Any product that may be evaluated in this article, or claim that may be made by its manufacturer, is not guaranteed or endorsed by the publisher.

## Footnotes

1
https://www.oie.int/app/uploads/2021/03/book-amr-ang-fnl-lr.pdf

2
https://clsi.org

3
https://eucast.org

4
https://itol.embl.de

## Supplementary Material

Supplementary Figure 1 Genetic environment of *mcr-1* in the 17 *mcr*-positive *Escherichia coli* (MCRPEC) strains. The direction of arrows indicates the direction of transcription.
