# Supplementary figures and images for: Prevalence and Characteristics of mcr-1-Producing Escherichia coli in Three Kinds of Poultry in Changsha, China
Source: Front Microbiol. 2022 Apr 7;13:840520. doi: 10.3389/fmicb.2022.840520 (PMC9021793; doi:10.3389/fmicb.2022.840520)

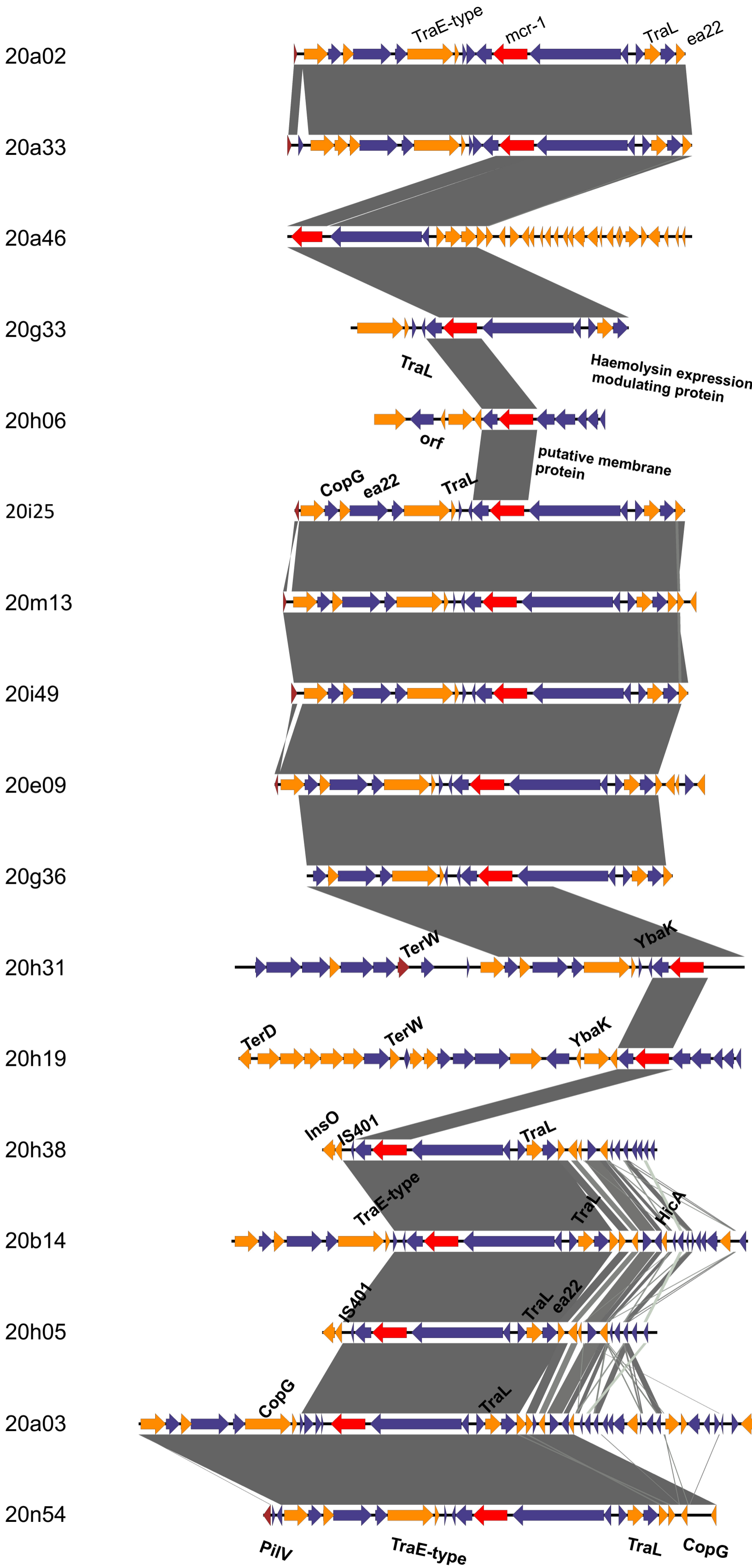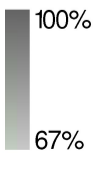

Supplement: Supplementary Figure 1 — Genetic environment of mcr-1 in the 17 mcr-positive Escherichia coli (MCRPEC) strains. The direction of arrows indicates the direction of transcription. [file Image_1.pdf]
