# Supplementary material for: Prevalence and Characteristics of mcr-1-Producing Escherichia coli in Three Kinds of Poultry in Changsha, China
Source: Front Microbiol. 2022 Apr 7;13:840520. doi: 10.3389/fmicb.2022.840520 (PMC9021793; doi:10.3389/fmicb.2022.840520)
Supplement: Supplementary file 2 [file Table_1.docx]

Table S1. The isolation rate of MCRPEC in poultry farm

| Farm | Source | Number | MCRPEC |
| --- | --- | --- | --- |
| liuyang 1 | broiler | 50 | 8.0%(n=4) |
|  | laying duck | 50 | 2.0%(n=1) |
|  | fly | 10 | 0 |
|  | sewage | 2 | 0 |
|  | drinking water | 2 | 0 |
|  | soil | 5 | 0 |
| liuyang 2 | laying duck | 50 | 0 |
|  | fly | 5 | 0 |
|  | sewage | 2 | 0 |
|  | drinking water | 2 | 0 |
|  | soil | 5 | 0 |
| liuyang 3 | laying duck | 50 | 0 |
|  | fly | 2 | 0 |
|  | sewage | 2 | 0 |
|  | drinking water | 2 | 0 |
|  | soil | 5 | 0 |
| liuyang 4 | broiler | 50 | 2.0%(n=1) |
|  | fly | 10 | 0 |
|  | sewage | 2 | 0 |
|  | drinking water | 2 | 0 |
|  | soil | 5 | 0 |
| liuyang 5 | quail | 50 | 0 |
|  | fly | 10 | 0 |
|  | sewage | 2 | 0 |
|  | drinking water | 2 | 0 |
|  | soil | 5 | 0 |
| liuyang 6 | broiler | 50 | 4.0%(n=2) |
|  | fly | 2 | 0 |
|  | sewage | 2 | 0 |
|  | drinking water | 2 | 0 |
|  | soil | 5 | 0 |
| liuyang 7 | broiler | 50 | 10.0%(n=5) |
|  | fly | 4 | 0 |
|  | sewage | 2 | 0 |
|  | drinking water | 2 | 0 |
|  | soil | 5 | 0 |
| liuyang 8 | broiler | 50 | 4.0%(n=2) |
|  | fly | 3 | 0 |
|  | sewage | 2 | 0 |
|  | drinking water | 2 | 0 |
|  | soil | 5 | 0 |
| changshaxian 1 | quail | 50 | 2.0%(n=1) |
|  | fly | 5 | 0 |
|  | sewage | 2 | 0 |
|  | drinking water | 2 | 0 |
|  | soil | 2 | 0 |
| changshaxian 1 | quail | 50 | 0 |
|  | fly | 5 | 20%(n=1) |
|  | sewage | 2 | 0 |
|  | drinking water | 2 | 0 |
|  | soil | 2 | 0 |
| total | - | 690 | 2.5%(n=17) |
